# A Review of Particle Therapy for Skull Base Tumors: Modern Considerations and Future Directions

**DOI:** 10.14338/IJPT-20-00083

**Published:** 2021-06-25

**Authors:** Eugen B. Hug, Maciej Pelak, Steven J. Frank, Piero Fossati

**Affiliations:** 1 MedAustron Ion Therapy Center, Wiener Neustadt, Austria; 2 MD Anderson Cancer Center, Division of Radiation Oncology, Houston, TX, USA

**Keywords:** skull base tumors, chordoma, carbon ion therapy, particle therapy, proton beam therapy

## Abstract

Skull base tumors constitute one of the established indications for particle therapy, specifically proton therapy. However, a number of prognostic factors, practical clinical management issues, and the emerging role of carbon ion therapy remain subjects of active clinical investigation. This review summarizes these topics, assesses the present status, and reflects on future research directions focusing on the management of chordomas, one of the most aggressive skull base tumors. In addition, the role of particle therapy for benign tumors of the skull base, including pituitary adenoma and acoustic neuroma, is reviewed.

## Introduction

Primary tumors of the skull base are rare overall. However, if cancers of the head and neck region with secondary invasion of the skull base are included, for example, paranasal sinus cancers or primary intracranial tumors with location in the skull base (ie, meningiomas), the numbers rise, and familiarity with delivery of high-dose radiation to the skull base becomes a necessity for the practicing radiation oncologist.

For many years, treatment of skull base tumors has been considered one of the established indications of particle therapy [1]. Yet, no randomized trial or level I evidence has ever been provided. Rather, its initial success has been based on an unprecedented leap of faith in technology combined with a keen sense of biologic knowledge of disease as well as anticipated normal tissue tolerances by the pioneers of particle therapy in the 1970s and 1980s. At a time when the most common treatment-planning techniques were based on 2-dimensional calculations, particle facilities were among the first to routinely use 3-dimensional planning systems.

For the first time this permitted the reliable calculation of partial critical organ-at-risk (OAR) high-dose delivery and to distinguish between, for example, surface and center dose of the brainstem rather than quoting doses to OARs as a single crude number.

Whereas blocks were routinely used to limit radiation to the brainstem or optic chiasm and optic nerves to 45 to 50 Gy, particle pioneers permitted partial volume doses of 60 to 64 Gy. Standard fractionated doses to the skull base beyond 70 Gy were virtually unheard of at the time. Throughout the 1980s and 1990s, despite relatively limited patient numbers, the pioneering treatment experience using this revolutionary technology and treatment-planning approach in combination with a daring vision to venture into dose escalation in a critical anatomic region fraught with risks of severe side effects, if not death, summarily led to an early acceptance of particle therapy for skull base tumors [2].

The skull base is an anatomically complex and functionally critical area. It is also the site of origin of several mesenchymal tumors, the most common being chordoma and chondrosarcoma, with rarer occurrences of osteosarcoma, hemangiopericytoma, malignant peripheral nerve sheath tumor, and so on. Because of its anatomical peculiarities, oncologically adequate wide surgical resections can almost never be performed in the skull base. Maximum safe surgical resection, followed by high-dose radiation therapy, is considered the standard of care. Several excellent reviews provide a complete summary of published data on treatment strategy and outcomes [1, 3–12].

In this article we will not attempt to systematically review once again the existing literature. Rather, we will try to focus on specific critical aspects that may be helpful in routine clinical decision making and for the design of future clinical research activities related to chordomas and chondrosarcomas. In the second part we will discuss the role of particle therapy for benign skull base tumors using the example of pituitary adenomas and acoustic neuromas.

## Chordomas and Chondrosarcomas of the Skull Base

Both chordomas and chondrosarcomas are rare tumors with an incidence of approximately 0.1/1 000 000 population per year for chordomas. Chondrosarcomas make up approximately 0.15% of all intracranial and 6% of skull base tumors [6]. Since chordomas arise from the embryonal notochord, they can only arise in the skull base from the clivus itself. In contrast, chondrosarcomas occur most commonly laterally from the petroclival junction and the petrous bone. The morphology differs as chordomas are greyish and gelatinous in appearance, whereas chondrosarcomas tend to show calcification. This is important from a surgical point of view. Chordomas have the potential for treatment with suctioning in addition to a resection; therefore, the tumor could potentially be removed from anatomic areas that are difficult to access. In contrast, the tendency for calcification in chondrosarcomas can make a surgical resection rather difficult, especially when in proximity to critical normal structures.

Most chordomas and chondrosarcomas are of grade 1 or grade 2 differentiation, although both entities may be high grade [12].

### Clinical Features and Prognostic Risk Factors

What should be the expectations for overall outcome assuming individual best standard of care for chordomas and chondrosarcomas? Bohman et al [13] analyzed the Surveillance, Epidemiology, and End Results (SEER) database for 1983 through 2009. This included 416 patients with chordomas and 269 with skull base chondrosarcomas. For chordomas, overall survival rates were 65% at 5 years and 32.3% at 10 years. The corresponding rates for chondrosarcomas were 81.8% at 5 years and 49.5% at 10 years. This included patients treated with surgery only, patients treated with surgery plus postoperative radiation, and patients treated with definitive radiation therapy, including some treated with protons. Given that local failures precede death by an average of 2 to 3 years, the 5-year local control rates, although not reported, were likely in the order of 45% to 50% for chordomas.

A number of practical management issues are relevant for treatment management recommendations at multidisciplinary tumor boards [14–20]. If one accepts that particles are indicated for midsize tumors of the skull base with limited gross residual disease after an adequate surgical debulking or subtotal resection, then the controversies are about the indications and efficacy of particle therapy at either side of the spectrum: (1) postoperatively for small or visibly gross totally resected disease and (2) for large unresectable disease with compression of such critical structures as optic chiasm and brainstem.

Publications that stratify the analysis according to tumor size point toward local control rates of approximately 90% to 95% after postoperative particle therapy without evidence of gross residual disease or for small tumors up to approximately 20 to 25 cm^3^ in size [21]. Presently, no data are available indicating a local control difference between postoperative proton therapy for no residual disease or small volume disease. Local control rates of 90% to 95% long term after proton therapy need to be compared with surgery alone. Unfortunately, such surgical series are difficult to find. Even larger surgical series include in their patient population an often unidentified subgroup of patients who have received postoperative particle therapy. Wang et al [14] reported on 238 chordoma patients primarily undergoing surgical resection alone. Visibly gross total resection was accomplished in 11.8% and near total resection in 54.2% of patients. Those 2 groups were combined as patients with “marginal resection.” Overall survival and progression-free survival rates at 5 years were 76% and 45%, respectively. These data are in contrast to use of particle therapy for which repeatedly local control rates >90% are reported (see Table 1).

**Table 1.** Results of proton therapy for skull base chordoma.

**Institution**	**Year of publication**	**No. of patients**	**Mean prescribed dose (GyRBE)**	**Dose per fraction (GyRBE)**	**Median follow-up months**	**5-year local control (%)**	**5-year overall survival (%)**	**Late toxicity grade 3 or more**
LLUMC [2]	1999	33	65–79	1.8-2	33	59	79	7%
Tsukuba [22]	2004	13	72	2	69	46	67	3 patients
CPO [23]	2005	88	67	1.8/-2	31	54 (4 year)	81	nr
PSI [24]	2009	41	73.5	1.8	38	81	62	4 patients
University of Florida [25]	2014	33	77.4–79.4	1.8	21	86 (2 year)	92 (2 year )	0%
PSI [26]	2016	151	74	1.8-2	50	76	86	8%
CPO [27]	2018	106	68.4–73.8^a^	1.8	61	75	88	7%
HIBMC [28]	2018	11	57.6–74.0	2.0–3.6	71	80	73	2 patients
CNAO [29]	2020	70	74	2	44	84	83	11%

**Abbreviations:** LLUMC, Loma Linda University Medical Center (USA); CPO, Centre de Protonthérapie d'Orsay (France); nr, not reported; PSI, Paul Scherrer Institute (Switzerland); HIBMC, Hyogo Ion Beam Medical Center (Japan); CNAO, Centro Nazionale di Adroterapia Oncologica (Italy).

a Photon + protons. Dose prescription according to residual gross tumor volume.

It remains a matter of debate if one should routinely offer immediate adjuvant particle therapy in patients with no identifiable residual disease after surgery. One can justify it by quoting likely local control rates of 90% to 95%, but this has to be counterbalanced with the risks of severe (grade 3 or higher) side effects that will likely be in the 2% to 5% range.

As a counterpoint, in the analysis by Wang et al [14], an increasing number of surgeries negatively affected the chances of overall survival and progression-free survival as well as treatment for primary versus recurrent disease. Summarizing those factors, it appears that high-dose postoperative proton therapy would be indicated in patients for gross total resection and near total resection, given that they would remain in the more favorable prognostic category [14].

Many patients are referred for unresectable disease or after partial resection or prior resections with continued growth of residual disease—many of which have in common that critical normal structures are compressed by the tumor: brainstem, optic chiasm, and/or optic nerves. Because evidence indicates that chordomas and chondrosarcomas require dosages in excess of 74 GyRBE or 68 GyRBE, respectively, for a realistic chance of long-term tumor control, then the problem arises that abutment or compression of critical OARs results in underdosage of a significant portion of a chordoma or chondrosarcoma. Most centers experienced in proton therapy will not exceed tolerance dosages of 64 Gy to the surface of the brainstem or 60 Gy (defined as maximum dose or small volume dose) to the optic chiasm and/or optic nerves [12]. Indeed, 5-year local control rates of these large tumors are likely in the range of 50% to 60% (see references in Table 1). One accepts a small percentage of underdosage in the case of a small area of abutment. However, particle therapy based on protons clearly has its limitations in patients who present with a large unresectable skull base tumor that longitudinally compresses the brainstem in various locations, leading to a very unfavorable broad and deep shoulder in the dose volume histograms of gross tumor coverage. In these cases, a careful reevaluation of surgical debulking options is mandatory. If debulking is definitively ruled out, particle therapy remains the best option.

Is there any evidence that high-dose radiation therapy will improve outcome? Most recently, collaborators at Duke University reviewed the National Cancer Database on the treatment of 1478 chordoma patients, including 567 patients with skull base location [15]. Detailed analysis not only pertained to surgical results but also included a meta-analysis of radiation therapy techniques and radiation dosages delivered. The primary-outcome parameter was overall survival” A subgroup of advanced radiation therapy techniques was defined that included 432 patients treated with proton therapy, stereotactic radiosurgery ,or intensity modulated radiation therapy. The proton beam cohort comprised more than 50% of this group, that is, 243 patients. A significantly improved outcome by advanced radiation therapy compared with standard external beam radiation therapy was noted. On multivariate analysis for high-dose advanced radiation therapy, the 2 parameters of high dose (>65 Gy) and advanced radiation therapy technique were superior to conventional external beam radiation therapy. It can be assumed that the vast majority of patients in the group of proton beam radiation therapy received a dose >65 Gy.

Is sex a predictive factor? This issue was first raised by the MGH group with female patients having decreased local control rates [21]. Other centers were not able to confirm this. According to the retrospective analysis by Wang et al [14] on 238 patients, male versus female, and in its majority a surgical series of patients, sex was not found to have a predictive value.

Another interesting management question frequently discussed at tumor boards is that of watchful waiting in patients with small- or medium-volume disease. Taking this question a step further raises the issue of salvage therapy after proton failure. In other words, what are the risks that, due to watchful waiting after initial diagnosis and surgery, progressive disease will move the patient from the good-risk to poor-risk category, that is, having a lower likelihood of gaining local control.

What about salvage after radiation failure? The initial publication dates back to 1995, when Fagundes et al [30] reported an exceedingly poor survival of patients after failure of proton therapy, that is, long-term salvage was basically not possible. Those data have been recently confirmed by the collaborators at the Paul Scherrer Institute in Switzerland [19]. They analyzed 71 patients with recurring chordoma of the skull base after proton therapy (patients treated between 1997 and 2015). In this relatively modern series using various modalities of salvage, that is, surgery, chemotherapy, and state-of-the-art radiation therapy, the disease-specific survival after disease recurrence following proton therapy had a median of 3.9 years. The median overall survival was only a dismal 3.4 years. In summary, this supports the concept of instituting proton therapy at the time of optimum chance to gain local control because survival after failure is typically not long term [19].

## Established Treatment Strategy

In this section, we limit the analysis to chordoma as a paradigmatic example of the most critical issues. Maximum safe surgical debulking followed by high-dose radiation therapy is considered the treatment of choice for skull base chordoma [31].

Surgical resection can be conducted with open craniotomy or endonasal endoscopic approach. A relatively recent meta-analysis could not detect significant differences between the 2 approaches [24]. This equivalence was recently confirmed in several retrospective comparisons [14, 17, 33]. After surgical resection, high-dose radiation therapy is therefore indicated and can improve overall survival and local control, and particle therapy is considered the radiation modality of choice [15, 17, 31, 34].

### Results of Particle Therapy

Both proton therapy and carbon ion radiation therapy have been successfully used in the treatment of skull base chordoma. Excellent results have been reported with both modalities with limited (but still not negligible) severe late toxicity and high probability of local control. These results are summarized in Table 1 for protons and in Table 2 for carbon ions.

**Table 2.** Results of carbon ion radiation therapy for skull base chordoma.

**Institution**	**Year of publication**	**No. of patients**	**Mean prescribed dose (GyRBE)**	**Dose per fraction (GyRBE)**	**Median follow-up months**	**5-year local control (%)**	**5-year overall survival (%)**	**Late toxicity grade 3 or more**
GSI [35]	2004	44	60	3	32	74 (4 year)	86 (4 year)	0%
GSI [36]	2014	155	60	3	72	72	85	0%
HIBMC [28]	2018	13	57.6–74.0	2.0–3.6	71	92	100	2 patients
NIRS [37]	2019	34	60.8	3.8	108	77	93	4 patients
CNAO [29]	2020	65	70.4	4.4	44	71	82	11%

Abbreviations: GSI, Gesellschaft für Schwerionenforschung (Germany); HIBMC, Hyogo Ion Beam Medical Center (Japan); NIRS, National Institute of Radiological Sciences (Japan); CNAO, Centrbackoffice@medaustron.ato Nazionale di Adroterapia Oncologica (Italy).

In the past 15 years, different institutions have consistently reported local control rates in excess of 70%. These results have been possible thanks to a continuous refining of the treatment technique. A detailed review of technical aspects is beyond the scope of this article; It is, however, important to acknowledge that, beside the nominal dose distribution, such parameters as beams number and orientation play a key role. Plan robustness must be considered in order to safely apply particle therapy in the skull base and the risk of overshooting into OARs or of underestimating the relative biological effectiveness of the distal high linear-energy transfer part of the particle beam must always be taken into consideration.

Protons have a longer treatment history and have been applied in multiple academic centers over the past 20 to 25 years. Having long-term follow-up data, in terms of tumor control, survival, and side-effects profile, makes protons the standard against which carbon ions have to be measured. With relatively favorable outcomes demonstrated for proton therapy, the introduction of carbon ions in this setting must be carefully scrutinized Applying this concept to chondrosarcomas of the base of skull, the first issue is the excellent local control, even for large tumors. In particular, for small and medium-size tumors, a switch to carbon ions would primarily require a justification based on reduction of long-term side effects. Although there are no solid data that tie an admittedly low local failure rate to size, applying carbon ions in the setting of a prospective study appears justifiable.

For small-volume chordomas with local control rates in excess of 90% long term, we would not routinely apply carbon ions. The major management issue for small chordomas remains temporal lobe toxicity. If carbon ions would likely lower the incidence of temporal lobe parenchymal toxicity, then a case could be made.

As outlined earlier, medium-size chordomas with unfavorable geometry and large-volume unresectable tumors have lower control rates; thus, given the encouraging reports from National Institute of Radiological Sciences [37], we would apply carbon ion therapy in a prospective setting. At present, it is not possible, by analyzing these retrospective data, to conclude anything about the relative merits of protons and carbon ions. A multicenter randomized trial combining carbon ion centers with similar treatment philosophy and similar dose determination systems would be desirable, and an appropriate patient stratification is a mandatory prerequisite.

The rationale to expect an improvement in large-tumor local control with carbon ions is based on the increased efficacy of their high linear-energy transfer against the hypoxic and radioresistant tumor clones. This advantage can be verified only by clinical results. The potential advantage of carbon ions in midsize tumors compressing the brainstem and/or chiasm derives from their sharper penumbra and smaller spot size. Ultimately, clinical data are also needed to confirm this advantage; it is, however, possible to estimate this advantage by plan comparison and make personalized decisions accordingly.

## Future Developments

In the large majority of particle therapy series, gross tumor volume correlated with local control, and patients with small residual disease had a better outcome [22, 24, 26–29, 36, 37]. Moreover, several series demonstrated a correlation between compression of brainstem and/or optic pathways and outcome [26, 29, 34]. Finally, target coverage emerged as another factor strongly predictive of outcome [23, 27, 29, 37].

Despite the extreme rarity of skull base chordoma, a wealth of data has been made available, and the individual institutions have to be commended for their efforts. While contemporary outcomes can be considered satisfactory, there is still room for improvement in terms of increasing local control and reducing toxicity. To achieve these goals, a more in-depth analysis is needed focusing on prognostic/predictive factors and multidisciplinary treatment planning. A first major limitation is that no staging system is used for skull base chordoma. The TNM staging for bone sarcoma is hardly applicable for the skull base, and the same holds true for the older Enneking staging system. The only attempt to stratify skull base chordoma patients according to tumor extension at diagnosis is the Sekhar grading system for cranial chordomas (SGSCC) [38]. This is a numeric score taking into consideration tumor size; invasion of brainstem, dura, and blood vessels; and primary versus recurrent disease. Its original purpose was to predict the difficulty of the surgical procedure; it has, however, been shown to correlate with final outcome (local control, progression-free survival, and overall survival) in a large series of patients receiving proton therapy at the Paul Scherrer Institute [18].

The treatment of skull base chordoma is surgical debulking plus postoperative radiation therapy, and these 2 steps should be planned and analyzed simultaneously. From the surgical point of view, it must be recognized that the same procedure has 2 quite different goals: debulking and separation. To confirm the real value of tumor burden reduction, it would be advisable to report not only gross tumor volume but also presurgery volume. Moreover, the real impact of gross total resection versus minimal residual disease (10 mL) versus small residual disease (25 mL) should be analyzed. Even more crucial for final outcome is the role of decompression/separation. There is a direct link between the quality of surgical resection and the possibility of achieving adequate target coverage with radiation therapy. The SGSCC penalizes vascular encasement; however, carotid artery involvement can be relatively easily salvaged by particle therapy. In the future, SGSCC should be revised to account for not only surgical outcome but also final outcome after surgery plus radiation therapy.

This information should drive the planned surgical resection. Particle centers are still a scarce resource, and international and even intercontinental referral is still quite common. In clinical practice, a crucial unsolved issue is the role and indication for a second surgical debulking before irradiation. A second debulking is almost never requested for residual disease in the cavernous sinus. On the contrary, there are other common scenarios in which a second debulking could be advantageous. One example would be residual lateral macroscopic disease at the petroclival junction that compresses the brainstem and was not resected with a transnasosphenoidal approach but could be resected with an open craniotomy. Another example would be residual macroscopic disease around the basilar artery in which the surgeon does not attempt to open the dura. Failure to achieve a theoretically possible brainstem decompression has a major impact on outcome, but this can only be quantitatively analyzed by examining surgical and radiation therapy data together, taking into account the initial presentation, the surgical technique used, and the specific expertise of the surgical team.

A paradigm shift is needed from surgery followed by radiation to upfront joint treatment planning. This can be achieved only through stronger cooperation between the radiation oncologist and neurosurgeon and by mutual education. Ultimately, a radiation oncologist dealing with the skull base must be familiar with the pros and cons of different neurosurgical accesses, and the neurosurgeon has to acquire knowledge of OAR constraints and achievable dose gradients.

Another critical issue is tailoring particle therapy (especially in terms of dose, fractionation, and particle selection) according to adequate patient stratification. Escalating total dose, using a larger dose per fraction, and using carbon ion instead of protons might improve local control. None of the three approaches is, however, at present based on adequate evidence. In a recent article from the Italian center Centro Nazionale di Adroterapia Oncologica [29], proton therapy with 2 GyRBE per fraction was used in favorable cases (small residual gross tumor volume) and carbon ions with a high dose per fraction (4.4 GyRBE) was instead prescribed to patients deemed at higher risk. This selection was done according to clinical judgement. Interestingly in this series local control of patients with minimal residual disease (<10.4 mL) receiving protons was 100%.

Existing data strongly suggest the need for better patient stratification. Treatment deintensification should be explored in the favorable subgroup in the attempt to reduce the still non-negligible toxicity. On the other hand, more aggressive particle therapy should be explored for high-risk patients. In patients with residual brainstem compression despite maximal surgical debulking, treatment intensification should also include a careful prospective escalation of brainstem dose constraints at least for small subvolumes. Comparison of retrospective series and even prospective trials without adequate patient stratification may not be adequate to evaluate the optimal particle-therapy strategy.

The role of systemic therapy in the treatment of chordoma is at present rather limited. As well described in a recent review [39], several prospective trials are ongoing to test specific molecularly targeted drugs. The indication is at present limited to advanced disease, and patient selection through mutations analysis is recommended. Even more preliminary are the data on the role of immunotherapy in chordoma [39]. Despite extreme scientific interest, it is not foreseen that systemic therapy will play a major role in the treatment of localized skull base chordoma in the near future.

### Pituitary Adenoma

Pituitary adenomas comprise a range of functionally and prognostically distinct tumors arising from normal pituitary gland cells. Surgery, considered primary management, serves a therapeutic and a diagnostic measure: it removes the tumor while usually sparing the remaining normal gland, and it provides detailed data on microscopic features, including hormonal receptors, p53, and MIB-1 expression status, which determine prognosis and further management [39]. In daily clinical practice, radiation therapy is not applied in adjuvant settings but is rather reserved for recurrent or progressive disease in which no surgical intervention is possible or accepted by the patient. Therefore, the patient group eventually referred to radiation oncologists mostly presents with surgical complications (typically in the form of single or multiple pituitary hormonal deficits) or with tumors that have recurred multiple times [40]. Considering these risk factors, the reported local control rates after fractionated stereotactic photon irradiation [41–43] well exceeding 90% are very satisfactory. The studies used total radiation doses in the range of 45 to 54 Gy. Similar doses were applied by groups investigating the efficacy of proton beam therapy. The local control was comparable: Wattson et al [44] achieved a 98% local control rate in 140 patients with a median follow-up of 43 months, while Ronson et al [45] reported no radiologic failures in their cohort of 47 patients followed for a median of 47 months. These results demonstrate the success in negatively selected patients; most of whom had recurrent macroadenomas with a frequently high proliferation index and recurrence after repeated resections. These results also indicate that the rationale for proton therapy in the treatment of pituitary adenomas is more driven by reduction in unwanted side effects than by improvement in target coverage. One advantage of particle radiation compared with volumetric modulated arc therapy is its ability to precisely shape the dose around spherical volumes. For carefully selected patients in whom there is a clear border between the normal gland and tumor, the normal pituitary would be identified as a new OAR and could benefit from a reduced radiation dose with an approach similar to that employed in radiosurgery [46]. The outcomes of studies by groups who analyzed the gross risk of newly induced hypopituitarism in patients were inconclusive (8% to 88%); however, there was a notable tendency for a higher incidence of hypopituitarism in series with longer follow-up. The results of the study by Ronson et al [45] look promising in this aspect, as they used higher doses (54 Gy for all patients) than other researchers and observed radiation-induced pituitary insufficiency in approximately one-fourth of patients. Due to the central tumor location, another potentially avoidable toxicity involves neurocognitive deficits, which would originate from deposition of low radiation doses to significant bilateral normal brain volume. Early evidence indicates the importance of bilateral sparing of these structures [47, 48] and demonstrates the dosimetric advantage of proton therapy over volumetric modulated arc therapy in that regard [49] for target volumes to the central base of the skull. It remains to be prospectively verified whether these data translate to significant difference in neurocognitive performance for pituitary adenoma. Lastly, long survival after radiation treatment of pituitary adenoma may translate to a non-negligible risk of secondary central nervous system malignancies [50], which is likely to be lower for proton therapy than photon irradiation [51]. The benefit of using proton therapy for fractionated irradiation is therefore expected to be most prominent for younger patients and those without hypopituitarism after the surgeries. Data are lacking for more aggressive pituitary tumors requiring a radiation dose in excess of 54 Gy; however, based on dosimetric properties of different radiation modalities and the widely adapted tolerance doses of surrounding OARs, we expect that emerging data will demonstrate a local control advantage for proton therapy for aggressively recurring macroadenomas.

### Acoustic Neuroma

Acoustic neuroma is a benign yet functionally burdensome tumor that is clinically associated with hearing deterioration, vertigo, and tinnitus. The treatment is generally first indicated by radiologic and/or clinical progression. Similar to the previously discussed pituitary adenoma, the treatment options include surgery and irradiation [52]. For schwannomas, the choice of modality is to a greater extent determined by pretreatment symptoms, tumor size, location (particularly regarding brainstem proximity), and patient preference. For tumors up to about 25 to 30 mm in diameter and with adequate (3 to 5 mm) brainstem clearance, a radiosurgical approach using stereotactice radiosurgery or external-beam radiotherapy has been reported to yield highly satisfactory results with long-term local control exceeding 90% [53]. The dose used in single-fraction radiation treatment has also evolved toward more conservative values of 12 to 14 Gy to achieve balance between best possible tumor response and acceptable rates of radiation-induced cranial neuropathies (trigeminal, facial, and vestibulocochlear nerve) [54]. Most series treating with similar doses report that about 50% of patients maintain serviceable hearing and very low toxicity from trigeminus/facialis not exceeding 6%. Patients usually prefer the logistic relief of single-fraction treatment over multiple weeks of fractionated irradiation.

Some acoustic neuromas are, however, first diagnosed with size and brainstem clearance exceeding the feasibility limits for radiosurgery. Fractionated irradiation is the treatment of choice for these patients, patients with progressive disease after previous treatment, and patients for whom surgery is not feasible or not accepted. The results of fractionated stereotactic radiation using photons are similar with regard to radiologic tumor control [55, 56], with a trend toward better sparing of functional hearing on the affected side (71% to 94%). The ability of the proton beam to stop at a defined point in the body and to deliver practically no relevant radiation dose in its direction afterward makes this type of treatment potentially attractive, in particular for clearly lateralized target volumes. For this reason–because of the proton beam's ability to precisely limit the dose to neighboring OARs (which would be cochlea and brainstem in this case)–the safety and efficacy of proton treatment for schwannomas was evaluated in a number of studies. Harsh et al [57], Weber et al [58], and Vernimmen et al [59] reported outcomes with stereotactic proton therapy delivered across 1 to 3 fractions. While maintaining optimal local control above 95%, the hearing preservation rate of 33% to 42% was suboptimal to radiosurgery and fractionated photons.

Another area of concern was the relatively high risk of cranial nerve injury, which happened in 9.5% to 10.4% of patients. These predominantly involved sensoric or motoric dysfunction of the facial nerve; less commonly affected was the trigeminal nerve. A proportion of patients (1.9% to 4.7%) had grade 3 neuropathies that required surgical procedures due to functional and cosmetic deficit. Conventional fractionation appears to be a safer and equally efficient method to apply protons. The published results [60] maintain local tumor control at the level of previously described modalities (92% to 97% depending on the dose used), yet demonstrate a more favorable toxicity profile (hearing preservation 44% to 64%, 2% risk of cranial nerve V or VII neuropathies). Note, however, that long-term results are not yet available for the spot-scanning proton therapy technique using conventional fractionation, which would be technically most feasible to precisely reduce the radiation dose to the cochlea and consequently further reduce the risk of hearing deterioration. The added benefit of lower integral dose to the brain remains the rationale for fractionated protons compared with photon fractionated stereotactic radiation therapy, similar to the findings for pituitary adenoma. Patients who will likely benefit the most from this treatment are younger patients with large tumors and functional hearing; but, because of the long expected survival, secondary malignancy and cognitive deterioration risks have to be considered. Based on the current level of evidence, the patients likely to benefit the most from particle therapy for acoustic neuroma are younger patients who are not candidates for radiosurgery for technical reasons and/or want to preserve functional hearing.

## Conclusion

Proton therapy for skull base malignancies has been practiced for decades. Practitioners have used proton therapy to pioneer necessary dose escalation to target volumes while pushing the limits of contemporarily accepted dose tolerances for critical normal structures. Early on, it has contributed to our understanding of partial volume irradiation of critical normal structures and provided essential clinical data. For various radioresistant tumor histologies, it has demonstrated its ability to provide permanent local control and, thus, in many instances, cure for small to moderate-size tumors. Carbon ion therapy holds the promise of significantly improving local control for large-volume unresectable disease. In general, a combined approach with surgery first for maximum debulking followed by proton therapy is preferred, if realistic, and consistent with a moderate toxicity profile. In addition, removing tumor components that abut or compress critical structures, such as the brainstem, optic chiasm, or optic nerves, remains one of the main goals of surgery. For histologically benign, but locally aggressive, skull base tumors, particle therapy provides an excellent opportunity. Assuming similar chances of local control by using similar radiation dose schemata, the focus in the coming years will be on quality of life, neurocognitive outcomes, and long-term preservation of sensory functions.
